# Recent Advances on Fluorine Chemistry

**DOI:** 10.3390/ijms25158251

**Published:** 2024-07-28

**Authors:** Mikhail Yu. Moskalik

**Affiliations:** A.E. Favorsky Irkutsk Institute of Chemistry of the Siberian Branch of the Russian Academy of Sciences, Favorsky Street, 664033 Irkutsk, Russia; moskalik@irioch.irk.ru

The purpose of this Special Issue is to showcase the latest findings in fluorine chemistry. Fluorine is a unique element due to its introduction as a substituent in various classes of chemical compounds. What makes fluorine so unique is its special properties that affect the reactivity of molecules. These properties are both polarizing and steric, and they have a surprising impact on the chemical and physical characteristics of substances. The features play a crucial role in every stage of research related to fluorine-containing structures. They influence the development of methods for synthesizing and analyzing fluorine compounds. They also guide the study of how these compounds interact with living systems, revealing their biochemical mechanisms of action. Moreover, they inform the research trends on the synthesis and structure of new materials that incorporate fluorine. The series of manuscripts submitted for this Special Issue explore various aspects of lanthanide fluoride research. These investigations focus on the use of lanthanide (YF_3_, LaF_3_, LnF_3_) contraction and the chemical and structural implications. Additionally, the manuscripts delve into the thermodynamic mechanisms behind the formation of two-component materials, RF_3_ (R = for Pm, Sm, Eu or Gd), with controllable properties in a binary system. One of the manuscripts in the Special Issue discusses the process of removing fluoride ions from high-concentration semiconductor industry wastewater. This method aims to recover high-purity CaF_2_ crystals. Another manuscript describes the reactions of alkenes with a new fluorine-containing nitrene precursor phenyl-N-triflylimino-*λ*^3^-iodane, PhI=NTf. The reactions hold promise for developing innovative applications in various fields.

The field of fluorine chemistry is constantly evolving, driven by new synthetic methods and analysis techniques. In recent years, there has been a surge in the development of innovative fluorination methods. One of the methods is visible-light-mediated fluorination, which allows reactions to occur at room temperature and under mild conditions. The approach enables fluorination processes to be carried out selectively in precisely defined positions, without the need for large amounts of catalysts that are often required in traditional fluorination methods. Selectfluor or NFSI serve as fluorinating reagents in this process [1]. A recent study involved the demonstration of a method for the selective fluorination of the (sp^3^)-CH bond in the presence of elemental iodine. In the reaction, NEt_3_·3HF acts as the fluorinating agent. The combination of iodine’s remarkable properties as a catalyst and amidyl-radical formation allows for the selective fluorination of C(sp3)-H bonds on the tertiary carbon atom [2]. The oxidative fluorination of Te- or S-containing substrates in the presence of TCICA/KF has seen significant advancements in recent years. The method has been developed to a high level of efficiency and selectivity, making it a valuable tool for the organic synthesis and chemistry of elements [3]. In particular, allylsilanes react with NEt_3_·HF to produce 2-fluoro-3-silylpropan-1-ols, which are important intermediates in organic synthesis. These fluorohydrins can be easily functionalized due to the presence of a silicon-containing group, making them versatile starting materials for various synthetic approaches. The method for synthesizing fluorohydrins involves the opening of an oxirane ring (pre-formed) under the action of NEt_3_·HF. The reaction occurs at room temperature, demonstrating high regioselectivity due to the β-effect of silicon. Additionally, a one-pot epoxidation and epoxide ring-opening has been developed for certain substrates, further expanding the applications of this approach. One of the most remarkable aspects of these reactions is the absence of desilylation under the action of fluoride anions, which highlights the stability of the silicon-containing groups in the presence of fluoride ions under the reaction conditions [4].

Methods for the selective addition of fluorine to multiple bonds are being actively developed. This task is challenging in organic synthesis. Recently, a method for the fluorination of alkenes with AgF was demonstrated. The reaction involves using *N*-bromodialkylamines and AgF. Fluoride not only serves as a source of fluorine, but also acts as a catalyst initiating the SET process. The reaction is versatile and can be applied to a wide range of substrates, including styrenes and non-activated alkenes (terminal, 1,1-disubstituted, or internal alkenyl derivatives). The reactions result in the formation of tertiary alkylamines containing fluorine atoms, which have important applications in the synthesis of bioactive molecules and their fluorinated analogs [5]. The fluorination of allenes, resulting in the formation of synthetically valuable propargyl fluorides, is achieved in the presence of aryl iodides [6].

Aminofluorination reactions are also a promising area for future research. For example, aziridines can serve as a nitrogen source in the aminofluorination reaction, facilitated by tBu-XPhos as the fluorination agent [7]. 

The reaction between 3-methylene-1-(trifluoromethyl)cyclobutanecarbonitrile and tert-butyl-3-methylenecyclobutanecarboxylate yields analogs of γ-amino- and γ-hydroxybutyric acids. These compounds are important neurotransmitters and excellent precursors for the synthesis of neuroactive derivatives. The reactions occur with good diastereoselectivity [8]. Additionally, a method for fluorination to the benzene ring using Pd(OAc)_2_ and NFSI was described in the work [9].

In addition, classical methods of functionalization and heterocyclization play a significant role in fluorine chemistry. Methods for synthesizing and assembling heterocycles from fluorinated building blocks are being actively developed [10,11,12]. When iodosylbenzene is present, BF_3_·Et_2_O acts as a catalyst and fluorination agent for asymmetric nucleophilic fluorination. Enamides are employed as substrates to produce oxazines [13]. As an example, CF_3_-containing hydrogenated imidazothiazines were synthesized from readily available reagents under mild conditions. Moreover, the cyclization of 2-mercaptobenzimidazoles and β-CF_3_-1,3-enynes can be achieved through the action of bases such as DBU or KOH. Depending on the choice of the bases, the reaction leads to the formation of 3,4-dihydro-2H-benzo[4,5]-imidazo[2,1-b][1,3]thiazine with CF_3_ substituents (for DBU) or 4H-benzo[4,5]imidazo[2,1-b][1,3]thiazine (for KOH) [14].

Fluoroalkylation is another promising field in the chemistry of fluorine compounds. Although the methods of biocatalysis in fluorination reactions are still developing, there are already successful examples of their use. This approach is particularly important for medical chemistry and biochemistry. For instance, a recent study demonstrated the method of fluoromethylation of small molecules using fluorinated SAM (S-adenosyl-S-(fluoromethyl)-L-homocysteine). This approach utilizes the ability of halide methyltransferases to form fluorinated SAM from S-adenosylhomocysteine and fluoromethyliodide [15]. In the work [16], an amazing method for forming the tetrafluoroisopropyl group ((CF_2_H)_2_CH) was shown using commercially available CF_2_HSO_2_Na. The tetrafluoroisopropyl group is widely used in organic synthesis, medicinal chemistry, and surface modification methods. The reaction of CF_2_HSO_2_Na with alkenes or alkynes, induced by visible light, forms α-tetrafluoroisopropylcarbonyl derivatives or cyclopentanones. This approach to C1-C3 fluoroalkylation allows polyfluorinated derivatives to be obtained [16]. (Trifluoromethyl)trimethylsilane (TMSCF_3_) is a highly reactive and efficient trifluoromethylation agent. However, reactions involving the simultaneous introduction of both TMS and CF_3_ groups at the same reaction center were not previously known. It was described how amides can be converted into α-trifluoromethyl-α-quaternary amines that contain a versatile and reactive silicon-containing substituent (TMS). The reaction is made possible by using a catalytic system based on Sm and SmI_2_ [17]. 
